# Specific Immunologic Countermeasure Protocol for Deep-Space Exploration Missions

**DOI:** 10.3389/fimmu.2019.02407

**Published:** 2019-10-11

**Authors:** George Makedonas, Satish Mehta, Alexander Choukèr, Richard J. Simpson, Gailen Marshall, Jordan S. Orange, Serena Aunon-Chancellor, Scott M. Smith, Sara R. Zwart, Raymond P. Stowe, Martina Heer, Sergey Ponomarev, Alexandra Whitmire, Jean P. Frippiat, Grace L. Douglas, Stephanie S. Krieger, Hernan Lorenzi, Judith-Irina Buchheim, Geoffrey S. Ginsburg, C. Mark Ott, Meghan Downs, Duane Pierson, Natalie Baecker, Clarence Sams, Brian Crucian

**Affiliations:** ^1^JES Tech, Houston, TX, United States; ^2^Laboratory of Translational Research “Stress & Immunity”, Department of Anesthesiology, Hospital of the Ludwig-Maximilians-University, Munich, Germany; ^3^Department of Nutritional Sciences, The University of Arizona, Tucson, AZ, United States; ^4^Department of Pediatrics, The University of Arizona, Tucson, AZ, United States; ^5^Department of Immunobiology, The University of Arizona, Tucson, AZ, United States; ^6^University of Mississippi Medical Center, Jackson, MS, United States; ^7^Department of Pediatrics, Columbia University, New York, NY, United States; ^8^NASA Johnson Space Center, Houston, TX, United States; ^9^University of Texas Medical Branch, Galveston, TX, United States; ^10^Microgen Laboratories, La Marque, TX, United States; ^11^Department of Nutrition, International University of Applied Sciences Bad Honnef, Bad Honnef, Germany; ^12^Institute of Biomedical Problems, Russian Academy of Sciences, Moscow, Russia; ^13^KBR, Houston, TX, United States; ^14^Stress Immunity Pathogens Laboratory, Lorraine University, Nancy, France; ^15^Infectious Disease Group, J. Craig Venter Institute, La Jolla, CA, United States; ^16^Duke Center for Applied Genomics and Precision Medicine, Durham, CA, United States

**Keywords:** space immunology, immune countermeasures, Herpesvirus reactivation, stress management, immune surveillance

Historically, serious illness of astronauts on orbit is rare, however clinical episodes requiring therapeutic intervention have occurred during International Space Station (ISS) missions at a noteworthy rate (1, 2). Persistent exposure to the space environment exacerbates perturbations to the immune system (3). In support, the NASA “twins” study—an evaluation of a crewmember during a 1-year ISS mission—revealed significant changes between in-flight and non-flight time points in the gene expression patterns of several immune response pathways, DNA methylation patterns of genes that regulate T cell responses, and the signatures of plasma cytokines, to promote during spaceflight decreased cellular responsiveness and increased inflammation (4). Because future deep-space exploration missions will endure for an unprecedented amount of time, with increased magnitude of mission-associated stressors, it is reasonable to expect a higher incidence of morbidities. Previously, we published a comprehensive review of *potential* countermeasures to obviate the immune “problem” associated with spaceflight. Now, we present a *specific and personalized* immune countermeasure prescription for prospective astronauts embarking on deep-space voyage (Table 1).

**Table 1:** Specific immune system countermeasure (CM) protocol for exploration space missions; suitable for validation in ground-analog and orbital spaceflight conditions.

**Mission phase**	**CM category**	**Specific CM**
**Pre-mission**	Screening	• Herpesvirus serology • Clinical history (particularly allergy, dermatitis, etc.) • Immune function profile
	Vaccination	GSK “Shingrix”
**In-mission** (Continuous)	*Full mission duration. Best options to counteract spaceflight-associated immune dysregulation*	
	Nutrition/Supplementation	Functional food enriched diet^*^ Probiotic Vitamin D
	Exercise	$\begin{array}{l} \text{“Adequate” aerobic}\\ \text{“Adequate” resistive} \end{array}\;\}\;\begin{array}{l} \text{Maintained at levels}\\ \text{comparable to ISS} \end{array}$
	Stress Management	Stress relieving breathing exercises (20–40 min/day)
	Medications	Anti-histamine/Fexofenadrine^**^ (Personalized) Valacyclovir^***^
**In-mission** (Scheduled)	Monitoring	$\begin{array}{l} \text{Virus PCR}\\ \text{Virus serology}\\ \text{WBC/differential}\\ \text{Soluble measures}\\ \text{(IL-6, CRP)} \end{array}\;\}\;\begin{array}{l} \text{Development of in-}\\ \text{flight laboratory}\\ \text{instrumentation} \end{array}$
**In-mission** (As Needed)	*[Periodic use or personalized countermeasures. Surgeon will address immediate clinical problem via “treatments.” The following options are countermeasures to rectify the underlying immune “problem” with implementation based on either in-mission monitoring or appearance of symptoms. Determination will be case specific, related to either chronic LVR, or acute LVR with clinical presentation]*	
	Medications^****^	Valganciclovir hydrochloride (if not Block 1) SC IL-2 G-CSF/Filgrastim SC Polyclonal Immunoglobulin Cytomegalovirus Immune Globulin

* *Enriched for omega-3 fatty acids, antioxidant, flavonoids; with adequate nutrient content: energy, protein, vitamins, and minerals*.

** *Only true non-drowsy antihistamine*.

*** *Pending results of NASA funded ground study*.

**** *Conditioned storage and stability will be re-evaluated based on final vehicle/mission architecture*.

A *pre-mission “screen”* of a crewmembers' clinical history, strength/aerobic fitness, and immune function will inform a personalized in-mission treatment standard. For example, some ISS crews are asymptomatic whereas others use antihistamines throughout their missions (2). Also, viral sero-status will determine the need for induced, virus-specific immunity. Cumulative data from Space Shuttle and ISS missions reveal a direct correlation between mission duration and the frequency/magnitude of latent herpesviruses (EBV, CMV, VZV) reactivation (5). Thus, it will be imperative to ensure a crewmember's virus-specific antibody titers are maximal pre-flight. During a pre-mission screen, if a crewmember exhibits VZV shedding and/or sub-optimal VZV-specific adaptive immunity, then he/she will receive the latest VZV subunit vaccine (Shingrix, by GlaxoSmithKline).

The *standard, in-mission protocol* contains a robust regimen for stress management because deep-space exploration will heighten physical and psychological stressors, which impact immune function negatively (6, 7). Stress-relieving breathing and/or mindfulness/positive visualization exercises can counteract the negative effect of stress on immunity (6). Some ISS crewmembers have reported beneficial effects after performing these exercises. Also, these techniques are popular at McMurdo Station in Antarctica for relief from the stress associated with communal living in a harsh environment. The countermeasure protocol aims to maximize the health benefits of these stress-relieving interventions by mandating daily time periods (20–40 min) for mindfulness, positive/creative visualization, breathing exercises and mechanized pressure-point (or related) therapy, with or without biofeedback. Ground validation of this countermeasure should incorporate a sensitive immune readout of stress, like NK cell function.

*In-mission immune surveillance* is necessary to sustain immune competence. For example, a simple lymphocyte count after a solar particle event may communicate a need for immune intervention. There are many miniaturized/microfluidics/microgravity-compatible laboratory instruments (available and in development) to perform a basic complete blood count, and to quantify soluble proteins that could serve as reliable biomarkers of inflammation. Two such blood cell analyzers launched to ISS in 2019 for validation. Viral shedding may be assessed during spaceflight by screening saliva samples with microgravity-compatible nucleic acid amplification tests (e.g., isothermal methods or microfluidics PCR). In short, the availability of instruments with a small footprint to surveil the immune system in real-time is critical to ensuring the success of the countermeasure protocol.

An onboard supply of immune “boosting” medications and treatments will be available for as-needed use. Medicines such as polyclonal immunoglobulin (IG) and Interleukin-2 (SC) are true “immune countermeasures” designed to rectify immune decrements. Hyper-immune polyclonal IG may confer additional benefits by combating latent herpesviruses reactivation. Antivirals are well-tolerated and should be included to mitigate clinical risks from herpesvirus reactivation. An upcoming NASA study will validate continuous antiviral use during Antarctica winter-over. Other medications—beta-blockers to reverse a perceived Th2 shift during spaceflight, and anti-inflammatory medications—may be useful too.

Onboard ISS, crewmembers consume adequate nutrition, however for deep-space exploration the storage conditions and longevity of the missions may compromise the availability of fruits/vegetables and sources of omega-3 fatty acids. If these staples cannot be maintained, nutritional intake may turn inadequate, which could exacerbate already-present changes in immunity. Agencies will work to achieve optimized nutrition via a “functional foods” rich diet. In this regard, the countermeasure protocol includes probiotic and vitamin D supplementation (7). Clinical evidence suggests certain probiotics may be beneficial because they counteract some spaceflight-relevant illnesses: antibiotic-associated diarrhea, respiratory infections, and dermatitis. Therefore, for deep-space missions, consuming a combination of probiotics—including strains of *Lactobacillus acidophilus, Lactobacillus casei*, and *Bifidobacterium lactis*—should be implemented (8). An ongoing study by the Japanese space agency is investigating the effect of probiotic supplementation on the health of ISS astronauts; those results will inform any final countermeasures strategy.

The countermeasures regimen we define herein incorporates several medical and behavioral tactics physicians on Earth prescribe their patients with weakened and/or compromised immune systems: therapeutics, nutrient-enriched diet, regular exercise, adequate rest, and stress-relief. Our hypothesis is that implementing all of them, simultaneously, will maximize immune health. At a minimum, the immune benefits proffered by the countermeasure protocol will raise the threshold for pathogenesis significantly. We assume, (1) a mission itinerary that maintains adequate sleep and circadian rhythm, and (2) a level of nutrition, muscle strength, and cardiorespiratory fitness comparable to what is maintained on ISS. These assumptions may be invalidated by the relatively constrained habitable volume, resupply options, and environmental control to accommodate crew exercise protocols. Some of the countermeasure medications may be incompatible presently with the operational constraints (storage mass, stability, delivery) but we anticipate future development in hardware will render them acceptable. Similarly, medications that are experimental currently may become standard treatments by the time we embark on deep-space exploration, including checkpoint inhibitors to modulate the immune system; we will augment the protocol accordingly.

The next step is for NASA and international partners to validate the regimen at ground-analogs—Antarctica winter-over, Russian “Sirius” isolation project (9, 10)—and during imminent ISS flight studies. Ideally, the final countermeasure protocol will be personalized for every crew member based on a pre-mission stint at an analog station.

## Author Contributions

GMak and BC conceptualized and wrote the manuscript. GMar, JO, and GG contributed valuable insight regarding applied clinical medicine. SM, DP, and CS provided Herpesvirus care input. AC, J-IB, MH, SP, JF, and NB contributed professional counsel from the European Space Agency (ESA) perspective. SA-C provided astronaut experience with stress management while on orbit. MD provided exercise physiology intelligence. SS, SZ, GD, HL, and CO provided expertise for the nutritional and dietary consumption outlook. RJS, RPS, AW, and SK contributed expert counsel on space immunology and immune surveillance.

### Conflict of Interest

GM and SM are employed by company JES Tech. RPS is employed by Microgen LLC. AW, SK, and MD are employed by KBR. The remaining authors declare that the research was conducted in the absence of any commercial or financial relationships that could be construed as a potential conflict of interest.
